# Modality-specific organization in the representation of sensorimotor sequences

**DOI:** 10.3389/fpsyg.2013.00937

**Published:** 2013-12-11

**Authors:** Arnaud Boutin, Cristina Massen, Herbert Heuer

**Affiliations:** IfADo – Leibniz Research Centre for Working Environment and Human FactorsDortmund, Germany

**Keywords:** sensorimotor representation, stimulus modality, chunking, implicit/explicit processing, sequence learning

## Abstract

Sensorimotor representations of movement sequences are hierarchically organized. Here we test the effects of different stimulus modalities on such organizations. In the visual group, participants responded to a repeated sequence of visually presented stimuli by depressing spatially compatible keys on a response pad. In the auditory group, learners were required to respond to auditorily presented stimuli, which had no direct spatial correspondence with the response keys: the lowest pitch corresponded to the leftmost key and the highest pitch to the rightmost key. We demonstrate that hierarchically and auto-organized sensorimotor representations are developed through practice, which are specific both to individuals and stimulus modalities. These findings highlight the dynamic and sensory-specific modulation of chunk processing during sensorimotor learning – sensorimotor chunking – and provide evidence that modality-specific mechanisms underlie the hierarchical organization of sequence representations.

## INTRODUCTION

In daily life we are surrounded by multiple sources of sensory information (Robertson and Pascual-Leone, 2001). Our capacity to act on the external world by efficiently gathering and processing sensory information coming from different modalities (e.g., visual, auditory) is a fundamental aspect of human cognition, which constitutes the bedrock for coherent and skilled behaviors (see Conway and Christiansen, 2005). Understanding the ability to integrate and represent behaviorally relevant sensory information devoted to action production is a central issue in the sensorimotor control and learning literature (e.g., Robertson and Pascual-Leone, 2001; Abrahamse et al., 2009; see Abrahamse et al., 2010, for review; Boutin et al., 2010). Here we ask whether the organization of the internal representation of a sensorimotor sequence is affected by the modality – visual versus auditory – of the sensory signals.

Insights into how the brain represents sensorimotor skills are provided by sequence learning paradigms (e.g., Verwey, 2001; Verwey et al., 2010). These paradigms are highly suitable for the study of the organization of relevant environmental information for action production (e.g., Boutin et al., 2010). Learning of complex serial behaviors involves the binding of discrete, independent actions into unified sequences of actions, called motor chunks (e.g., Sakai et al., 2004, for review Verwey et al., 2010). It has been suggested that improved performance in the course of learning entails a gradual transition from a sequence of individual movements to the preparation and execution of one or more series of movements, which is the hallmark of chunk processing (e.g., Verwey et al., 2002; Rhodes et al., 2004). Several lines of evidence suggest that the resultant segmentation of the movement sequence reflects a hierarchical organization at the representational level (Wright et al., 2010). In theory, processing within a motor chunk is considered to be carried out automatically by the motor system, while processing between motor chunks is thought to be controlled by the cognitive system (e.g., Rushworth et al., 2004; Sakai et al., 2004).

Operationally, motor chunks are defined by certain characteristics of a response-time profile, where response times (RTs) are plotted as a function of the serial position of the responses within a sequence. The response-time profile is not only determined by the physical characteristics of the responses, such as the fingers used, and the transitions between responses, such as within-hand and between-hand transitions, but primarily by characteristics of the sequence representation. In particular, a long RT followed by one or more considerably shorter RTs marks the beginning of a chunk (Verwey et al., 2010). The organization of the sequence representation can go beyond the chunking of individual responses, with chunks becoming integrated into higher-level units, so that a hierarchical organization emerges (e.g., Povel and Collard, 1982; Rosenbaum et al., 1983; Koch and Hoffmann, 2000).

In previous studies of chunking and the hierarchical organization of sequence representation, mostly sequential key-press tasks with visual-spatial stimuli have been used (e.g., see Sakai et al., 2004, for review; Verwey et al., 2010; Wright et al., 2010). In addition, the sequence was typically constructed with an inherent and obvious organization (Koch and Hoffmann, 2000). The advantage of such sequences is that almost all participants adopt the a priori organization, as reflected by mean response-time profiles. In contrast, when the sequence does not adhere to an obvious organization, individual participants adopt individual organizations (Sakai et al., 2003). In the present study, we test whether individual organizations of a sequence, which is void of an obvious inherent organization, reflect the modality of the stimuli.

Sequences can be represented in terms of the stimuli, in terms of the responses, or in terms of stimulus-response (S–R) compounds (see Abrahamse et al., 2010, for review). The hypothesis that the organization of sequence representations might be affected by stimulus modality could be taken to imply a stimulus component of the representation. However, an effect of stimulus modality could also occur if only motoric features of the responses were represented. For instance, visual and auditory stimulus sets would in general have different S–R compatibility with the response set, as it is the case in the present experiment. These differences are likely to be associated with different variations of RTs across the sequential responses. The role of temporal factors, such as longer inter-stimulus intervals, for the organization of sequence representations is well known (e.g., Stadler, 1995). Thus, a regular pattern of RTs could shape the organization of the sequence representation, even if only motoric features were represented, and this shaping should be different for different response-time patterns associated with different stimulus modalities.

There are other differences, such as the spatial frame which is present for visual, but not for auditory stimuli. Such a spatial frame could support the organization in other units than the ones preferred without a frame. Consider the characterization of relational structures that can be used in organizing a sequence representation (example elements are 1 2 3 4), such as runs (1 2 3…), trills (1 2 1 2…), repetitions (1 1 1…), reflections (1 2 4 3…), and transpositions (1 2 3 2 3 4…; Restle, 1970). Without a spatial frame, for instance, reflections might be less conspicuous than with such a frame, which provides boundaries at which reflections could occur. More generally, biases toward certain organizations are likely to be different for spatial patterns of successive visual-stimulus locations and for “musical” patterns of successive auditory-stimulus pitches. Even without specific hypotheses on what these differences are, they should affect the individual organization of a to-be-learned sequence that is not dominated by an inherent and obvious organization.

In this study, we contrast two practice conditions with different modality-based S–R compatibility with the response set. Specifically, both practice groups are required to perform the very same sequence of motor responses. However, their practice conditions differ in terms of sensory stimuli (visual and auditory). That is, sequence production relies on different sensory-motor mappings in the two groups: visual-motor and auditory-motor. Hence, based on the assumption that sensory-based mechanisms contribute to sequence structuring and the formation of motor chunks, we hypothesize that practice of a motor sequence that does not convey any a priori hierarchical organization leads to the development of individual and modality-specific organizations of sequence representations.

## MATERIALS AND METHODS

### PARTICIPANTS

Thirty undergraduate students from TU Dortmund University participated in this study in exchange for course credit or 15…. They were randomly assigned to the “Visual” (*N* = 15; Mean age = 22.2 ± 1.9 years; six females) and “Auditory” (*N* = 15; Mean age = 23.2 ± 2.8 years; four females) practice conditions when arriving at the laboratory. All participants were right-handed according to the Edinburgh Handedness Inventory (Oldfield, 1971), had normal or corrected-to-normal vision, and were unaware of the specific purpose of the study. They gave written informed consent prior to participation in the experiment, which was conducted with the general approval of the local ethics committee.

### APPARATUS AND STIMULI

**FIGURE 1:**
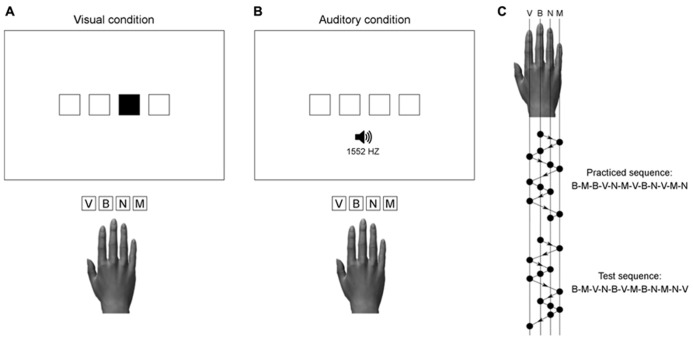
**Illustration of the stimulus-response mapping used in the visual (A) and auditory (B) practice condition.** Auditory stimuli with frequencies of 300, 675, 1552, and 3565 Hz, were respectively assigned to the response keys V, B, N, and M. The practiced and test sequences **(C)** were matched for number of movements per digit and two-finger transitions.

The display was viewed from a distance of approximately 50 cm. It showed four horizontally aligned squares presented white-on-black in the center of the screen. The squares were 2 cm wide and 2 cm high, spaced 1 cm apart. The mode of stimulus presentation (visual or auditory) was dependent on group assignment (see **Figure 1**). In the visual condition, a stimulus was one of the four squares filled green. In the auditory condition, stimuli were computer-synthesized tones of 50 ms duration, presented binaurally through stereo headphones. The four different tones used in this study had frequencies of 300, 675, 1552, and 3565 Hz. Stimulus presentation and response registration were controlled by custom-made programs using the Matlab® R2011b software (The MathWorks, Inc., Natick, MA, USA) and the Psychophysics Toolbox (Brainard, 1997; Pelli, 1997; Kleiner et al., 2007).

### TASK AND PROCEDURE

In the visual condition, participants were required to respond to visually presented stimuli (filled squares), which were spatially compatible with the response keys. In the auditory condition, participants were required to respond to auditorily presented stimuli, which had no direct spatial correspondence with the response keys. Each tone was assigned to a unique key on the computer keyboard, with the lowest pitch corresponding to the leftmost key (i.e., index finger) and the highest pitch to the rightmost key (i.e., little finger). **Figure 1** illustrates the S–R mapping used in both groups together with the practiced sequence and the test sequence.

In the auditory condition the S–R mapping was more difficult than in the visual condition (see Buchner et al., 1998). Therefore, participants assigned to the auditory condition underwent an initial *familiarization* phase of unrecorded trials in order to make them familiar with the (tone-key) S–R mapping, and to avoid high error rates during practice. They had to complete a 40-element sequence of randomly presented tones by depressing the corresponding keys. The instructions emphasized accuracy, which had to be above 85%. Only when participants had reached the pre-set learning criterion for the tone-discrimination performance, the practice phase started.

During the practice phase, participants were required to respond as rapidly and accurately as possible to a sequence of stimuli (visual or auditory) by depressing the appropriate response keys with their dominant right hand on a standard German QWERTZ keyboard. They held their right-hand index, middle, ring and little fingers on the response keys V, B, N, and M, respectively. Each practice trial began with the presentation of four empty squares. The first imperative stimulus was presented after a random foreperiod of 1–3 s (in 0.5-s steps). The response of the participant triggered the presentation of the next stimulus, and so forth until the end of the practice block. Each block consisted of 10 repetitions of a 12-element sequence. The time needed to produce the 120 key presses was shown as feedback at the end of each practice block for 5 s. The display was then erased, and the screen remained black for 20 s. Breaks were inserted between the 14 blocks that composed the practice phase to prevent fatigue. When participants were ready to proceed with the next practice block, they pressed any one of the four response keys.

To differentiate sequence learning from generalized practice effects, a test block with a new sequence of stimuli was presented after the end of practice. Performance with the 12-element test sequence served as a reference to determine sequence-specific learning of the practiced sequence (see Abrahamse et al., 2010). The order of stimuli in the test sequence was different from the practiced sequence, but the test sequence contained all of the two-finger transitions that composed the training sequence. Both in the practiced and test sequence the same key was not pressed twice in succession, and the same two-finger transition never occurred twice. No mention was made about the regularities in the order of stimuli.

After the test block participants were given a post-experimental free-recall test to evaluate their conscious awareness of the sequence, that is, their explicit knowledge. They were instructed to write down the sequential order of the 12 elements that composed the practiced sequence on a sheet of paper. Performance was scored by determining the number of serial positions for which the correct element was recalled.

### DATA ANALYSIS

Response time was defined as the time interval between stimulus onset and depression of the corresponding key. We designate the response times for the successive responses to the stimuli of the sequence as RT1, RT2 … RT11, and RT12. For each block of trials, we determined the error rate and the mean of all response times (neglecting error trials). For the analysis of response-time profiles, we computed the means of RT1, RT2 … RT11, RT12 from the 10 repetitions of the sequence in each practice block. These means were subjected to statistical analyzes as detailed in the results section.

## RESULTS

### MEAN RT AND ACCURACY DURING PRACTICE

**FIGURE 2:**
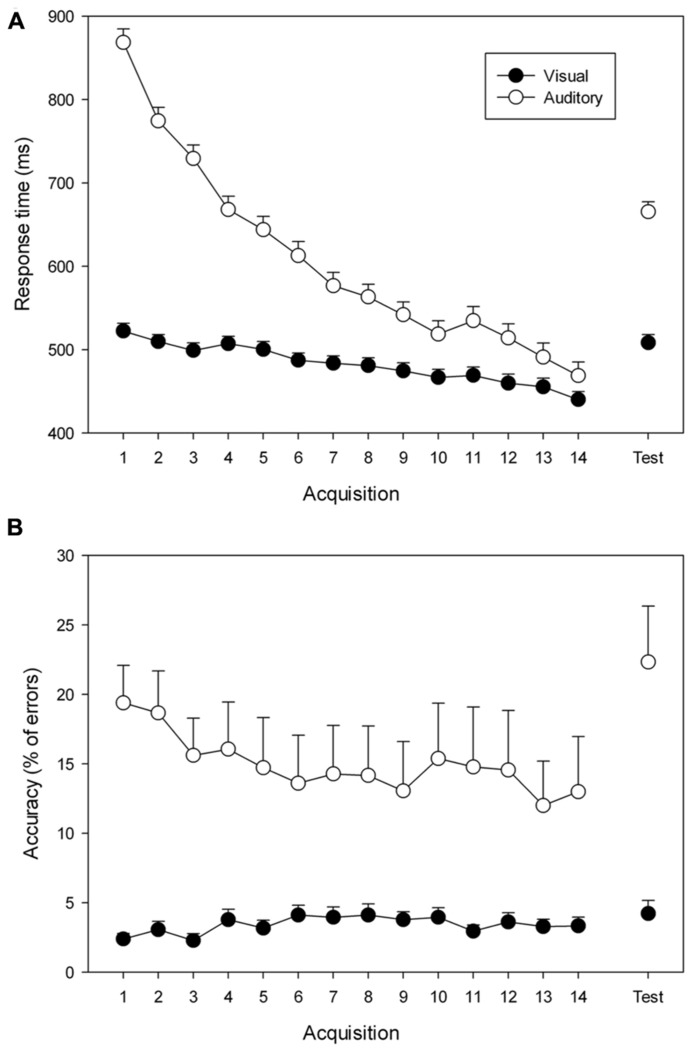
**Mean response times (A) and error rates (B) during practice (Blocks 1–14) and test (Test) for the visual (filled circles) and auditory (open circles) groups.** Error bars reflect the standard errors of the means.

Mean response times and error rates in the practice blocks are shown in **Figure 2**. They were submitted to separate 2 (Group: visual, Auditory) × 14 (Block: 1–14) ANOVAs with repeated measures on the factor block. When relevant, Duncan’s multiple range test was used to determine the specific effects contributing to the general ANOVA effects.

Analysis of RT during practice revealed a significant group × block interaction, *F*(13, 364) = 24.93, *p* < 0.001, $\eta_{p}^{2}$ = 0.47, reflecting group differences in the speeding-up of responses across practice blocks. *Post hoc* analysis indicated that both groups improved their performance from Block 1 to Block 14 (from 522 to 440 ms for the visual group, and from 868 to 468 ms for the auditory group; *ps *< 0.001). More specifically, participants of the visual group responded more rapidly than those of the auditory group from Block 1 (*p* < 0.001) to Block 3 (*p* < 0.01), but not from Block 4 (*p* = 0.07) to Block 14 (*p* = 0.73).

Mean error rate during practice amounted to 3.4% in the visual group and 16.8% in the auditory group. The analysis revealed a significant group × block interaction, *F*(13,364) = 2.85, *p* < 0.001, $\eta_{p}^{2}$ = 0.09, reflecting group differences in the evolving of error rates during practice. *Post hoc* comparisons detected a significant decline of the error rate in the auditory group across practice blocks (from 19.4 to 13.0 %; *p* < 0.001), but not in the visual group (from 2.4 to 3.3%; *p* = 0.57).

### SEQUENCE-SPECIFIC LEARNING

Mean response times and error rates in the test block are also shown in **Figure 2**. Differences to the last practice block reveal sequence-specific learning. RTs and error rates in the last practice block and the test block were analyzed in separate 2 (Group: visual, Auditory) × 2 (Block 14, Test) ANOVAs with repeated measures on the factor block. When relevant, Duncan’s multiple range test was used to determine the specific effects contributing to the general ANOVA.

Analysis of the response times revealed a significant group × block interaction, *F*(1, 28) = 7.43, *p* = 0.01, $\eta_{p}^{2}$ = 0.21. *Post hoc* comparisons indicated that both groups responded more rapidly on Block 14 than on the test block (440 and 508 ms for the visual group, *p* = 0.05; 468, and 665 ms for the auditory group, *p *< 0.001), which is the hallmark of sequence-specific learning. Moreover, the analysis indicated that the visual group was faster than the auditory group on the test block (*p* = 0.01), while no performance difference was observed on Block 14 (*p* = 0.64).

The analysis of the error rates revealed a significant group × block interaction, *F*(1, 28) = 9.95, *p* < 0.01, $\eta_{p}^{2}$ = 0.26. *Post hoc* analysis showed higher error rates for the auditory group on the test block than on Block 14 (respectively 13.0 and 22.3 %, *p* < 0.001), while no difference was observed in the visual group (respectively 3.3 and 4.2 %, *p* = 0.64).

### RESPONSE-TIME PROFILES

**FIGURE 3:**
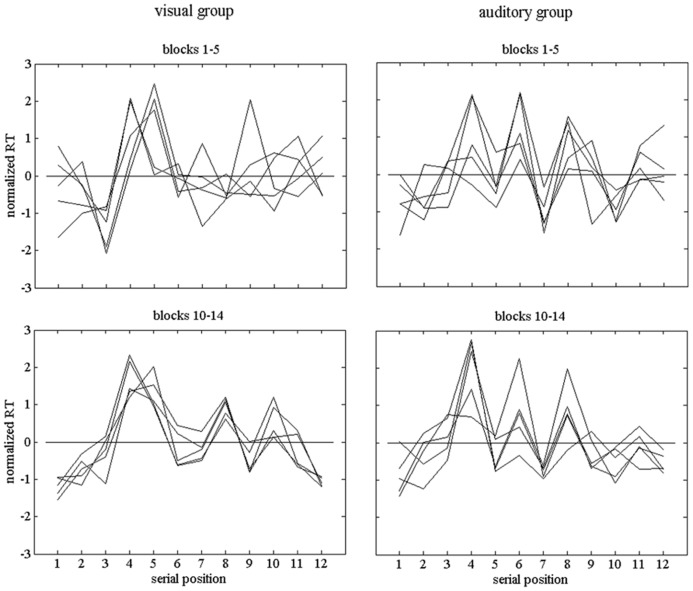
**Individual normalized response-time profiles of two participants, one of the visual group and one of the auditory group.** The upper row of graphs shows profiles during early practice (Blocks 1–5) and the lower row shows profiles during late practice (Blocks 10–14).

For each practice block, we computed the response-time profile across the 12 serial positions of the sequence. As expected, these profiles varied across participants. **Figure 3** shows example profiles of two participants, one in the visual and one in the auditory condition. Before plotting them, the profiles were normalized, that is, the deviations from the profile means were divided by the standard deviations of the RT1 … RT12 of the respective profiles. In addition, the profiles were grouped into those during early practice (Blocks 1–5) and those during late practice (Blocks 10–14). During early practice the decline of response time was faster than during late practice, and more marked changes of the profiles were expected.

We analyzed the profiles by way of computing distances between them. More specifically, we used 1 - *r* as a distance measure, with r as the correlation between two profiles. Geometrically, each profile can be conceived as a vector in 12-dimensional space. The correlation between two profiles is equivalent to the cosine of the angle between the vectors. Our measure of distance or dissimilarity is invariant against overall differences in response times between profiles as well as against different scalings of the RT variations across serial positions, but sensitive to shape differences, that is, to differences between the relative durations of the RTs in the profiles compared (see Heuer, 1984). The measure varies between 0 and 2 (respective correlations between 1 and -1), which corresponds to angles ranging from 0° to 180° between the respective vectors. The mean distances between pairs of profiles of blocks 1–5 (early practice) in **Figure 3** were 0.610 and 0.477 for the two participants in the visual and auditory group, respectively. For blocks 10–14 (late practice), they were 0.176 and 0.375, respectively.

**FIGURE 4:**
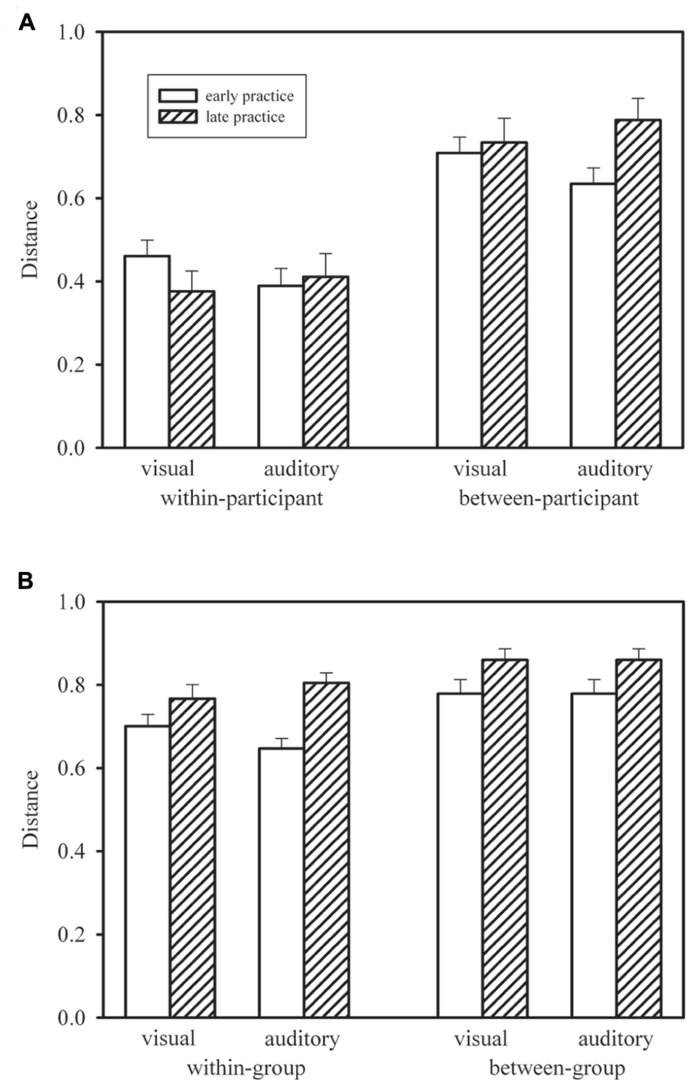
**Mean within-participant distances in the visual and auditory groups, during early and late practice, compared with re-sampled mean between-participant distances (A), and mean within-group distances compared with mean between-group distances (B).** Error bars show standard errors of the means. For between-participant distances **(A)**, error bars show standard deviations of the means of randomly re-drawn samples

**Figure 4A** shows the mean within-participant distances for early practice (Blocks 1–5; for each participant this was the mean of 10 distances computed between profiles from pairs of blocks 1–2, 1–3, 1–4, 1–5, 2–3, 2–4, 2–5, 3–4, 3–5, 4–5) and late practice (Blocks 10–14; for each participant this was the mean of 10 distances computed between profiles from pairs of blocks 10–11, 10–12, 10–13, 10–14, 11–12, 11–13, 11–14, 12–13, 12–14, 13–14) in the visual and auditory groups. We compared early and late practice by means of Wilcoxon signed-rank tests. Neither in the visual group, *T*(15) = 34, *p* > 0.10, nor in the auditory group, *T*(15) = 52,* p* > 0.20, was the distance between profiles significantly reduced during late practice. In addition, we compared the two groups by means of Mann–Whitney *U*-tests. These tests did not reach statistical significance, neither for early practice, *U* = 81, *p* > 0.10, nor for late practice, *U* = 122, *p* > 0.20.

In **Figure 4A**, the mean within-participant distances are compared with mean between-participant distances. In 1000 simulated random samples, we re-shuffled participants within groups for each practice block. For each of these pseudo “participants” we computed the same mean distances as in the case of the within-participant distances, but now – for each distance – the second profiles (from the later practice blocks) were from a randomly chosen different participant. These between-participant distances were considerably larger than the observed within-participant distances. In fact, for none of the simulated samples was the mean distance smaller than the corresponding observed mean within-participant distance. Thus, profiles were significantly more similar within than between participants. This is clear evidence of specific individual response-time profiles.

**Figure 4B** shows mean within-group (but between-participant) distances and mean between-group distances. For each participant and each block of trials we computed the mean distance to the profiles of the other 14 participants of the same group and of the 15 participants of the other group, and these means were averaged across Blocks 1–5 and Blocks 10–14. Thus, all distances were between profiles from the same practice block of different participants in same or different groups, whereas the distances shown in **Figure 4A** were always between profiles from different practice blocks of same or different participants within the same group. Note that the mean between-group distances shown in **Figure 4B** are identical for the two groups for mathematical reasons, whereas the standard errors are different. For each participant in each group, different distances entered the mean between-group distance, but across participants the set of distances was the same.

According to **Figure 4B**, within-group distances were smaller than between-group distances. For the statistical comparison by means of Wilcoxon signed-rank tests we collapsed both groups of participants. The within-group distances were significantly smaller both early in practice, *T*(30) = 3, *p* < 0.01, and late in practice, *T*(30) = 80, *p* < 0.01. In addition **Figure 4B** shows larger distances later in practice than earlier. The increase from early to late practice was significant both for within-group distances, *T*(30) = 52, *p* < 0.01, and between-group distances, *T*(30) = 83, *p* < 0.01, and it did not differ significantly between the two types of distance, *T*(30) = 186, *p* > 0.20. Thus, response-time profiles were more similar within each of the two groups who practiced with different stimulus modalities than between these two groups, and similarity declined in the course of practice, that is, individual profiles became more dissimilar.

### EXPLICIT KNOWLEDGE

The results of the free-recall test at the end of the experiment revealed that none of the participants was able to identify and report the entire practice sequence. They exhibited only fragmentary sequence recall with a mean of 5.2 ± 2.5 elements in the visual group, and a mean of 2.9 ± 1.3 elements in the auditory group. We compared the number of recalled elements between groups by means of Mann–Whitney *U*-tests. The statistical analysis indicated a significant difference between groups, *U* = 50, *p* < 0.01, revealing that the visual group expressed better explicit knowledge than the auditory group.

In an additional step we tested whether the difference in explicit knowledge could be critical for the smaller between-group (and between levels of explicit knowledge) than within-group (and within levels of explicit knowledge) similarity of response-time profiles. For this purpose we formed sub-groups with poorer and better explicit knowledge. For the visual group, there were nine participants with four or less correctly recalled elements and six participants with more than four correctly recalled elements; for the auditory group there were seven participants with two or less correctly recalled elements and eight participants with more than two correctly recalled elements. For each participant we contrasted the mean distance to the profiles of the other participants of the same group and the same sub-group with the mean distance to the other participants of the same group, but the other sub-group with a different level of explicit knowledge. Collapsed across all participants, early in practice the mean distances were 0.684 and 0.665 within and between sub-groups with different levels of explicit knowledge, respectively, and late in practice the mean distances were 0.792 and 0.767. According to Wilcoxon signed-rank tests, the differences were not significant, neither early in practice, *T*(30) = 168, *p* > 0.10, nor late in practice, *T*(30) = 161, *p* > 0.10.

## DISCUSSION

The present results reveal individual response-time profiles that become more different in the course of practice and, more importantly, a smaller variation of the profiles within than between the two groups who practiced with visual and auditory stimuli, respectively. Thus, the modality of the stimuli during sequence learning shapes the individual organization of the sequence representation, in particular the formation of motor chunks. These findings highlight the dynamic and sensory-specific modulation of chunk processing during sensorimotor learning and provide evidence of modality-specific mechanisms, which contribute to the hierarchical organization of sequence representations.

### SENSORY-BASED MECHANISMS FOR MOTOR CHUNKING

The present findings show that individuals organize sequence representations in different ways. Response-time profiles in successive practice blocks of the same person were more similar than response-time profiles in successive practice blocks of different persons. In addition, response-time profiles of different persons in the same practice blocks became more dissimilar in the course of practice. This elaborates observations according to which chunked representations of sequences are formed even when there is no a priori organization of the sequence, but these chunked representations differ between individuals (Sakai et al., 2003). In addition to the individual specificity of sequence organization, we show specificity for stimulus modalities. Thus, individual factors combine with the influence of the stimulus modality so that the response-time profiles at each stage of practice are more similar for persons for whom the stimulus modality is the same than for persons for whom the stimulus modality is different. In which way stimulus modalities shape the organization of sequence representations is currently as unknown as the individual factors that shape the organization. Only hypotheses are possible at the time being.

With respect to the underlying neural structures, there is considerable evidence that basal-ganglia circuits contribute to the formation of newly acquired skills in promoting the gradual structuring of the entire set of actions into ordered subsets (e.g., Graybiel et al., 1994; Graybiel, 1998; see Graybriel, 2008, for review). Intracranial recordings from the human basal ganglia provide evidence of an integrative role of this structure in the processing of sensory, cognitive, and motor information (see Bares and Rektor, 2001). Thus, considering that the basal ganglia also contribute to S–R learning (Graybiel, 1998), this opens the possibility that both visual and auditory sensory inputs participate in the shaping of the hierarchical organization of sequential sensorimotor behaviors.

The present study started with the hypothesis that the individual organization of sequence representations might be shaped by stimulus modalities. This hypothesis was confirmed, and it opens the question of how the effects of stimulus modalities come about. Regarding this question, we have no conclusive answer, but only a number of possibly contributing factors. The first type of factors relates to spatial characteristics of the visual stimuli that were not inherent to the auditory stimuli. This difference between stimulus sets is accompanied by a number of differences that might affect sequence representations. First, there is a difference in mean response time because of different levels of S–R compatibility. This difference could affect the degree to which stimulus characteristics are included in the sequence representation. Second, there are probably different patterns of delays between successive responses, which could shape the sequence representation. Third, the presence versus absence of spatial stimulus characteristics goes along with the presence of a spatial reference frame for visual stimuli, but not for auditory stimuli. Such a frame could modulate the conspicuity of certain relational structures such as reflections.

The second type of factors relates to the self-organizing tendencies that are inherent to sequences of visual and auditory stimuli. The spontaneous organization both of concurrently and successively presented stimuli, both visual and auditory, has been studied since the emergence of Gestalt psychology (e.g., Wertheimer, 1923), and it should be different for the stimulus sets of the present study. However, at present the spontaneous organizations of the stimulus sequences remain unknown because they are neither evident nor have they been studied empirically. Thus, although we provide firm evidence of modality-specific organization of sequence representations, the nature of the mechanisms involved remains as an unsolved problem.

### IMPLICIT LEARNING, EXPLICIT LEARNING, AND CHUNKING WITH VISUAL AND AUDITORY STIMULI

Sequential learning, and the accompanying chunking, is a multifaceted process with both implicit and explicit components (e.g., Nissen and Bullemer, 1987; Hikosaka et al., 2002; Robertson et al., 2004). The interplay between implicit (unconscious) and explicit (conscious) processing during sequence learning has long been the subject of theory and research (e.g., see Cleeremans et al., 1998, for review; Destrebecqz and Cleeremans, 2001; Robertson et al., 2004). Traditionally, while learners show improved performance when the same behavior is rehearsed, they often fail to exhibit verbalizable (explicit) knowledge about the acquired information (Willingham et al., 1989). This kind of learning is considered to be implicit (Cleeremans et al., 1998). Heretofore, most of the studies explored the extent to which awareness of the sequence relates to performance and learning (e.g., Destrebecqz and Cleeremans, 2001; Robertson et al., 2004). However, to the best of our knowledge, nothing is known about the extent to which explicit knowledge about the sequence affects the binding of the associate motor responses. We shall discuss both these relations of explicit knowledge to learning and chunking in turn.

A common indicator of (implicit) sequence learning is the difference between response times at the end of practice and in a test block in which the practiced sequence is replaced by a new one, often a random sequence. In the present study these learning scores were 68 ms in the visual group, but 197 ms in the auditory group. According to established standards, one would conclude that implicit learning was better with auditory than with visual stimuli. However, contrary to empirical evidence (e.g., Curran and Keele, 1993) and theoretical underpinnings (e.g., Keele et al., 2003), poorer learning scores in the visual group went along with better explicit knowledge, while better learning scores in the auditory group went along with poorer explicit knowledge. This finding nourishes doubts that the learning score is always an adequate measure of sequence-specific learning. These doubts are justified as soon as different levels of S–R compatibility are involved. The reasons are detailed in the following.

Consider initial performance in the present experiment. Response times were clearly faster in the visual group than in the auditory group because of different levels of S–R compatibility. At the end of practice this difference had almost disappeared, although effects of S–R compatibility typically survive extended practice periods, though they become smaller (Dutta and Proctor, 1992). Thus, in the present experiment the difference between the two groups would not have disappeared because the difference in S–R compatibility vanished as a result of practice, but because the stimuli, and thus the S–R mapping, became largely irrelevant for response selection. This is a consequence of acquiring a sequence representation and making use of it. The almost identical response times in the visual group and the auditory group at the end of practice could even be taken to suggest that the stimuli did not play any role whatsoever for response selection, and the sequence representations were based on response features only. This situation changes when a new sequence replace the practiced sequence. Then response selection is based on stimuli again, and different levels of S–R compatibility matter. Thus, the common learning score is affected by S–R compatibility, in particular by the differences between the simple mapping of sequence representations on responses and the high or low compatibility of S–R mappings. In general, learning scores should be larger when the S–R compatibility is low.

Regarding the question whether conscious awareness of the motor sequence influences sensorimotor chunking, the present data provide a partial answer. Response-time profiles of participants with similar levels of explicit knowledge were not more similar between participants than response-time profiles of participants with dissimilar levels of explicit knowledge. Accordingly, the dissimilar profiles of the two practice groups cannot be attributed to their difference with respect to explicit knowledge. Further, response-time profiles are not shaped by explicit knowledge in a way comparable to how they are shaped by stimulus modality. Thus, we tentatively suggest that the hierarchical organization of a sensorimotor sequence – sensorimotor chunking – is essentially an implicit process. This suggestion is tentative because explicit knowledge could modulate characteristics of response-time profiles for which our analysis is insensitive, such as the variability of response times across serial positions.

## CONCLUSION

We demonstrated that the sensory signals guiding task production have an important influence upon the process of skill acquisition. The structured response pattern that emerged through practice is dependent on the available sensory information, suggesting that sensory-based mechanisms mediate the formation of motor chunks. Findings account for an individual and modality-specific organization in the representation of sensorimotor sequences. Our results challenge purely motor-based accounts for chunk processing and lend support to the claim that sensory-based mechanisms underlie motor chunking – sensorimotor chunking.

## Conflict Of Interest Statement

The authors declare that the research was conducted in the absence of any commercial or financial relationships that could be construed as a potential conflict of interest.
